# Intracoronary allogeneic cardiosphere‐derived stem cells are safe for use in dogs with dilated cardiomyopathy

**DOI:** 10.1111/jcmm.13077

**Published:** 2017-03-15

**Authors:** Michael Taylor Hensley, Junnan Tang, Kathleen Woodruff, Teresa Defrancesco, Sandra Tou, Christina M. Williams, Mathew Breen, Kathryn Meurs, Bruce Keene, Ke Cheng

**Affiliations:** ^1^ Department of Molecular Biomedical Sciences and Comparative Medicine Institute North Carolina State University Raleigh NC USA; ^2^ Department of Cardiology The First Affiliated Hospital of Zhengzhou University Zhengzhou Henan China; ^3^ Department of Clinical Sciences North Carolina State University Raleigh NC USA; ^4^ Center for Human Health and the Environment North Carolina State University Raleigh NC USA; ^5^ Lineberger Comprehensive Cancer Center University of North Carolina Chapel Hill NC USA; ^6^ Joint Department of Biomedical Engineering University of North Carolina Chapel Hill and North Carolina State University Raleigh and Chapel Hill NC USA; ^7^ Eshelman School of Pharmacy University of North Carolina Chapel Hill Chapel Hill NC USA

**Keywords:** cardiosphere‐derived cells, dogs, dilated cardiomyopathy, stem cell therapy, allogeneic

## Abstract

Cardiosphere‐derived cells (CDCs) have been shown to reduce scar size and increase viable myocardium in human patients with mild/moderate myocardial infarction. Studies in rodent models suggest that CDC therapy may confer therapeutic benefits in patients with non‐ischaemic dilated cardiomyopathy (DCM). We sought to determine the safety and efficacy of allogeneic CDC in a large animal (canine) model of spontaneous DCM. Canine CDCs (cCDCs) were grown from a donor dog heart. Similar to human CDCs, cCDCs express CD105 and are slightly positive for c‐kit and CD90. Thirty million of allogeneic cCDCs was infused into the coronary vessels of Doberman pinscher dogs with spontaneous DCM. Adverse events were closely monitored, and cardiac functions were measured by echocardiography. No adverse events occurred during and after cell infusion. Histology on dog hearts (after natural death) revealed no sign of immune rejection from the transplanted cells.

## Introduction

Heart diseases remain the number one killer in western countries 1. Stem cell transplantation is a promising therapeutic strategy for acute or chronic cardiomyopathy, considering current treatments for human DCM usually involve intense drug regimen and/or invasive implantable devices 2. Small animal (rodent) studies are widely adopted for initial proof of concept 3. However, translation to human trials necessitates large animal studies (*e.g*. pigs, dogs). One dilemma is that many naturally occurring cardiomyopathies in humans cannot be modelled effectively in the laboratory. Interestingly, naturally occurring cardiomyopathy also affects the well‐being of domestic dogs. For instance, in Doberman pinschers, the DCM appears to represent a major cause of death 4. Dog and human DCMs share significant clinical similarities including but not limited to ascites, rhythm disruption, dyspnoea, syncope and sudden death 5, 6, 7, which inspired us to ask the following: can we use Doberman pinschers with DCM as a spontaneously occurring, clinically relevant large animal model of cardiomyopathy from which to translate cell‐based therapies for humans? The safety of adult stem cell therapy for heart diseases has been well established in humans 8. Over the last 7 years, our laboratory has been studying CDCs as a source to generate therapeutic cardiac progenitor cells for ischaemic heart diseases. A recent clinical trial indicates that CDC therapy benefits patients with mild‐to‐moderate myocardial infarction (MI) 9. The regenerative potential of CDCs has also been demonstrated in non‐ischaemic cardiomyopathy 10, 11, and thus, we hypothesize intracoronary CDC therapy can provide a safe and effective intervention for DCM; in addition, successful implementation of this intervention in a canine model of DCM will provide valuable insight into our ability to apply this technique to treat DCM in human beings.

## Materials and methods

### Derivation and culture of cCDCs

Canine CDCs were generated and expanded as described 3, 12 from myocardial specimens of a healthy male beagle dog 8, 13. Briefly, this tissue was surgically collected from the right ventricle of a 2‐year‐old canine beagle. An approximately 6 × 6 mm piece of myocardial tissue was separated and washed with phosphate‐buffered saline (PBS) (Life Technologies, Carlsbad, CA., USA). The tissue sample was then cut into smaller biopsy‐sized pieces and washed three times with PBS, followed by enzymatic digestion at 37°C in 5 mg/ml collagenase IV solution (Sigma‐Aldrich, St. Louis, MO, USA) for 5 min. Iscove's modified Dulbecco's medium (IMDM; Life Technologies) containing 20% foetal bovine serum (FBS; Corning, Corning, NY, USA) is then added to the sample to inactivate the collagenase. After that, the tissue samples were further minced into smaller tissue explants (~ 0.5 × 0.5 mm) before plating. Approximately 50 pieces of tissue explants were then placed onto a fibronectin‐coated plate with approximately 1.5 cm between each explant and covered with 2 ml of IMDM with 20% FBS overnight to aid the attachment. The cultures were maintained in 25–30 ml IMDM with 20% FBS, and media change was performed every other day. In about one week, cells started to outgrow from the tissue explants. Once these outgrowth cells are about 70–80% confluent, they were harvested by 5–10 min. of incubation with TrypLE Select™ (Life Technologies). The cells were then seeded into an ultra‐low‐attachment flask (Corning) at a density of 100,000 cells/cm^2^ and cultured in IMDM with 10% FBS. Phase‐bright canine cardiospheres (CSps) started to form in 3–7 days. Canine CSps were then collected from the low‐attachment flasks and replated onto fibronectin‐coated surface to produce adherent cCDCs. cCDCs were cultured in IMDM with 20% FBS media and passaged every 3–5 days.

### Flow cytometry analysis

cCDCs were characterized by flow cytometry as described 13, 14. Flow cytometry was performed on cCDCs using a FACSCalibur and LSR II (BD) and analysed using FLOWJO software (TreeStar, Carrboro, NC, USA). Cells were incubated with antibodies against CD105 (ab156756; Abcam, Cambridge, United Kingdom), CD90 (bd555595; BD Franklin Lakes, NJ, USA), CD117 (c‐kit, b5631; Abcam) for 60 min. Isotype‐identical antibodies served as negative controls.

### Release testing for cCDCs before injection into the dog patients

Gram stain testing (77730‐1KT‐F; Sigma‐Aldrich) was used for sterility testing for the final cell therapy product. Endotoxin testing was employed (N283‐06; Lonza, Basel, Switzerland) to ensure the cCDC product was endotoxin‐free. Cell viability and morphology were verified by trypan blue before infusion. Catheter cell retention testing was performed to ensure there was minimal cell loss between cell preparation and infusion into the heart chamber.

### Dog study protocol

All animal work is compliant with Institutional Animal Care and Usage Committee at North Carolina State University. Once client‐owned dogs showed signs of DCM and a left ventricular shortening fraction of <20% accompanied by LV dilatation, they were recruited from North Carolina to participate in the study. After informed consent signed by the dog owners, the dogs were randomized to either the CDC group or the control group (no cell therapy, only standard care). Dogs placed into the CDC group were given treatment 1 month after criteria were met. This study plan consisted of taking measurements over a twelve‐month period and contained five dogs in the CDC group (age 1768 ± 413 days) and three dogs in the control group (age 2366 ± 531 days). Subject information can be found on Table 1.

**Table 1:** Dog study design

	Screening/Baseline	Infusion	Day 1	1 month	2 months	6 months	12 months
Study day	Day −14 to Day 0	Day 0		Day 30	Day 60	Day 180	Day 360
Informed consent	X						
History/medication review	X	X		X	X	X	X
Adverse events assessment	X	X		X	X	X	X
Holter monitoring	X			X	X	X	X
Vital sign	X		X	X	X	X	X
BNP	X		X	X	X	X	X
Serum troponin	X		X	X			
1 EDTA (purple) Tube	X		X	X	X	X	X
1 Sera (red) tube	X		X	X	X	X	X
vWF screening	X						
Echocardiography	X			X	X	X	X
Intracoronary cell infusion		X					

### Infusion of cCDCs

Following premedication with butorphanol (0.3 mg/kg intramuscularly) and placement of an indwelling intravenous catheter, anaesthesia was induced with midazolam (0.2 mg/kg IV) and etomidate (0.35 mg/kg IV). Following routine endotracheal intubation, anaesthesia was maintained with a continuous rate infusion of fentanyl (0.2 μg/kg/min) combined with the lowest concentration of inhaled isoflurane needed to maintain a surgical plane of anaesthesia. Total anaesthetic duration for the procedures averaged 50 min.

The dog was moved to a right lateral recumbent position in the cardiac catheterization suite, and following aseptic preparation of the skin, a surgical cut down was performed in the right femoral triangle, exposing the right femoral artery. The artery was isolated and ligated distally with 3‐0 silk suture, and a 5 Fr × 7 cm Intradyn Braun vascular access sheath was introduced using modified Seldinger technique. A 5‐Fr. JR2.5 was advanced into the aorta under fluoroscopic guidance, and the right coronary ostium was engaged. A selective right coronary angiogram was performed utilizing a hand injection of 5 cc of nonionic contrast media Omnipaque (Y503; GE Healthcare, Little Chalfont, United Kingdom) to confirm catheter placement. The catheter was then gently flushed with 5 ml of LRS (07‐19‐63‐782; Baxter, Deerfield, IL, USA). 1 ml of PBS was then flushed into the right coronary artery over 30 sec., followed by a 5 ml suspension of stem cells over 5 min., followed by another 2 ml of PBS over an additional minute. The right Judkins catheter was then removed. A 5‐Fr. left Judkins catheter (JL3.5, 80 cm long) was then advanced under fluoroscopic guidance to engage the left coronary ostium, and a selective angiogram was performed with a hand injection of 5 ml of Omnipaque to confirm the catheter position. In a fashion identical to the right coronary injection, the catheter was gently flushed with 5 ml of LRS. Two millilitres of PBS was then flushed into the left main coronary artery over 30 sec., followed by a 10 ml suspension of stem cells over 5 min., followed by another 4 ml of PBS over an additional minute. Following the last injection of PBS, the catheter was removed, the catheter sheath introducer was removed from the artery, and the artery was double‐ligated with 3‐0 silk suture. The cut down incision was closed routinely, and recovery from anaesthesia was uneventful.

### Echocardiography measurement

Dogs were manually restrained, and all echocardiographic studies were performed using a Phillips IE‐33 echocardiographic system with simultaneous ECG. Standard imaging planes were obtained 15, and all data were captured digitally for offline analysis at a digital workstation. For this pilot safety study, simple M‐mode measurements of left ventricle end‐diastolic and end‐systolic dimensions (LVEDD and LVESD, respectively) were obtained from the right parasternal short‐axis image at the level of the chordae according to recommendations set by the American Society of Echocardiography 16. The average of three measurements from different sinus cardiac cycles was obtained. Fractional shortening (FS) was calculated from the M‐mode echocardiographic images as (LVEDD—LVESD/LVEDD) × 100%. As another indicator of cardiac function, per cent wall thickness (%WT) was determined using (end‐systolic wall thickness ‐ end‐diastolic wall thickness)/end‐diastolic wall thickness × 100% 17.

### Histology

Hearts were collected from canine patients after natural death and washed with PBS to remove excess biological fluids. Samples were either frozen in OCT compound (Tissue‐Tek) or placed in formalin and later processed into paraffin blocks. Heart sections (5 μm thick) were prepared from paraffin blocks. Haematoxylin and eosin stains were performed on samples (MHS1; Sigma‐Aldrich).

### Fluorescence *in situ* hybridization (FISH)

To detect the male donor cells in the female recipient heart, we perform Y‐chromosome fluorescence *in situ* hybridization (FISH). Two hundred nanograms from each sample was labelled using nick translation to incorporate one of three fluorochromes, Spectrum Red/Orange/Green dUTP (Vysis, Downers Grove, IL, USA). Typically, 25 ng of each of five differentially labelled probes was pooled and precipitated in the presence of 15 μg of sonicated genomic dog DNA as competitor. Chromosome preparation, probe hybridization and post‐hybridization washes were performed as described previously 18, 19. Chromosomes were counterstained in 80 ng/ml 4′, 6‐diamidino‐2‐phenylindole (DAPI) and mounted in antifade solution (Vectashield; Vector Laboratories, Burlingame, CA, USA). Images were acquired and processed using a multicolour FISH workstation comprising a fluorescence microscope [Olympus BX61 (Shinjuku, Tokyo, Japan) with zero shift, narrowband filters] CCD camera (CoolSnapHQ, Photometrics, Tuscon, AZ, USA) both driven by dedicated software (SmartCapture 2.3.1 Digital Scientific, Cambridge, UK). The digital image of each DAPI‐stained metaphase spread was processed using a high‐pass spatial filter to reveal enhanced DAPI bands. Clones were assigned to a chromosome region according to the DAPI‐banded nomenclature of Breen *et al*. 18, 19.

### Statistical analysis

Results are presented as mean ± S.D. unless specified otherwise. Comparisons between any two groups were performed using two‐tailed unpaired Student's *t*‐test. Comparisons among more than two groups were performed using one‐way anova followed by post hoc Bonferroni correction. Differences were considered statistically significant when *P* < 0.1.

## Results

### Generation of cCDCs

**Figure 1:**
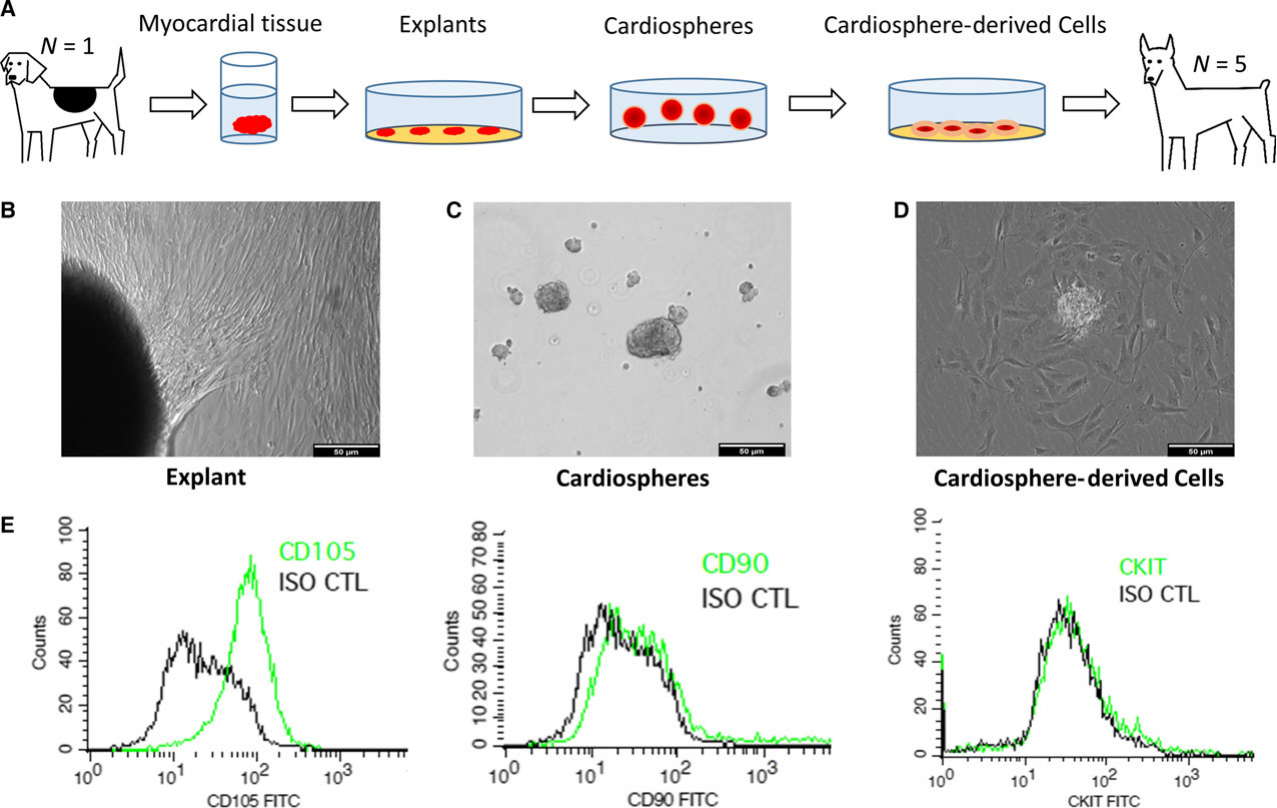
Derivation and culture of canine CDCs. (**A**) Schematic diagram showing the derivation of canine CDC a of Beagle dog. (**B**) Outgrowth cells from plated myocardial tissues. (**C**) Cardiosphere formation in suspension culture. (**D**) CDCs dissociated from cardiospheres. (**E**) Expressions of CD105, CD90, ckit by flow cytometry in canine CDCs. Scale bars = 50 μm in all images.

Using a three‐stage ‘adhesion–suspension–adhesion’ culture process (Fig. 1A), we derived CDCs from the myocardial tissues of a male Beagle dog donor. Both phase‐bright and stromal‐like cells started to outgrow from the canine heart tissue explants in a week after plating onto fibronectin‐coated surfaces. Those outgrowth cells become confluent in ~2–3 weeks (Fig. 1B). When seeded on low‐attachment surfaces (to discourage cell attachment), the outgrowth cells spontaneously aggregate into three‐dimensional canine cardiospheres (Fig. 1C). When replated onto a fibronectin‐coated surface, the cardiospheres dissociated into single cells which we termed CDC (Fig. 1D). One biopsy‐sized canine heart tissue can generate 50–200 million of passage 0 CDCs. Flow cytometry analysis (Fig. 1E) reveals that cCDCs were positive for CD105, similar to human CDCs.

### Intracoronary infusion of CDCs in Doberman pinscher with DCM

**Figure 2:**
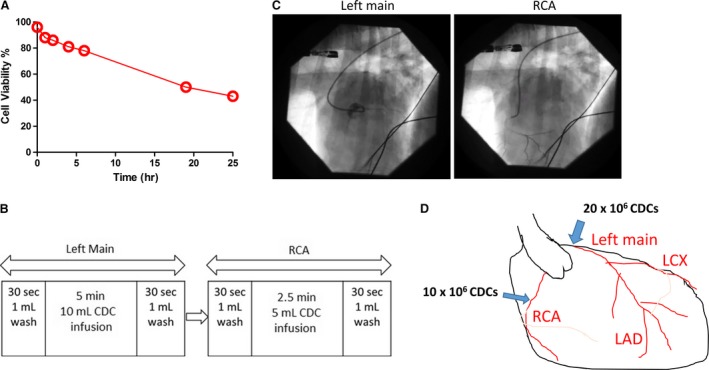
Cell infusion procedure. (**A**) Viability of CDCs stored at 4°C. (**B**) Intracoronary cell infusion design. (**C**) Coronary angiography showing catheter placement in left main and RCA. (**D**) Schematic diagram showing the infusion dose.

Catheter testing performed before infusion verified a low amount of cell loss through delivery, with >98% of cells passing through the catheter. Canine CDCs were harvested by TrypLE Select, placed in PBS with 100 U/ml heparin and stored at 4°C. Cell viability was >80% for up to 5 hrs (Fig. 2A) under this storage condition. The cells were infused with a regimen that was similarly applied in a previous human trial (Fig. 2B) 8. Coronary angiography (Fig. 2C) shows the placement of the infusion catheter in the patient's left main and right coronary artery (RCA). The infusion dose in each artery was described in Figure 2D.

### Effects of CDC therapy on cardiac functions of canines with DCM

**Figure 3:**
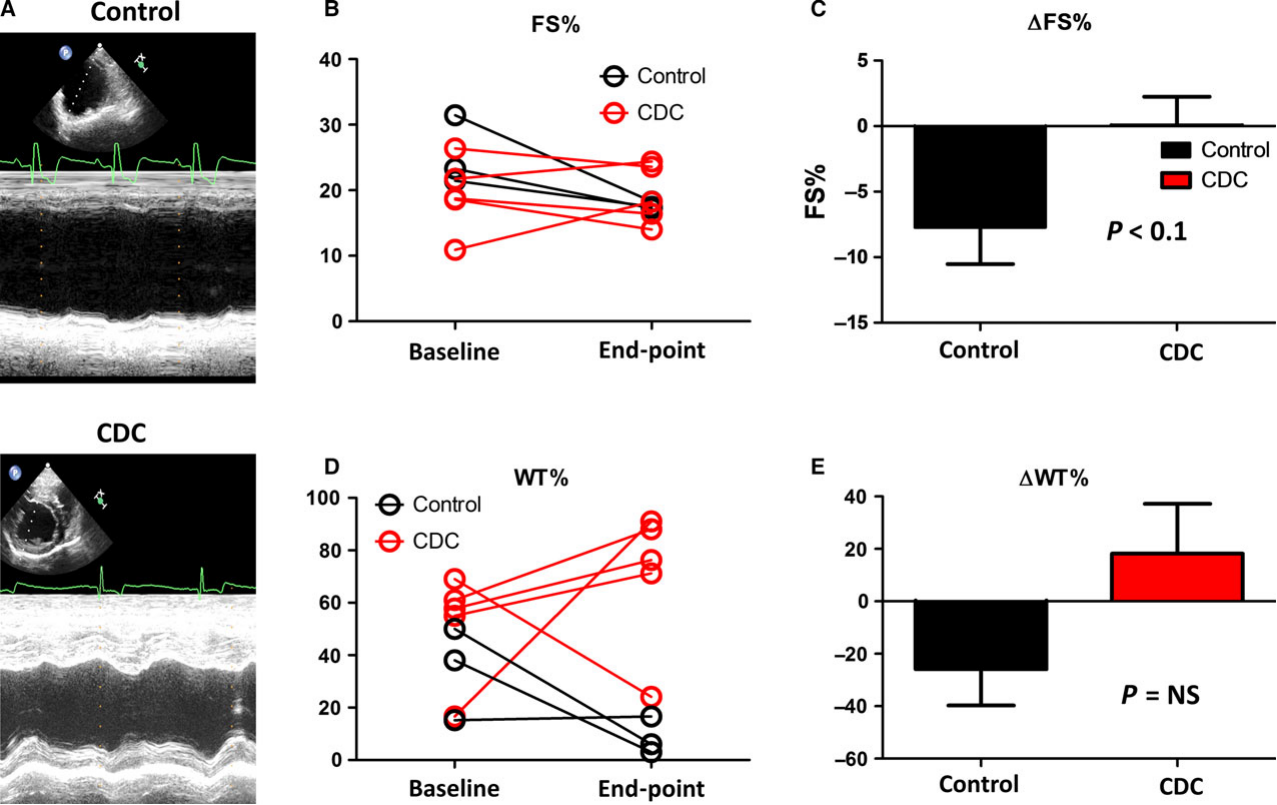
Effects of CDC therapy on cardiac function. (**A**) Representative echocardiography images showing control and CDC‐treated dogs at end‐point. (**B**) Fractional shortening (FS%) comparison between control and CDC groups. (**C**) Treatment effects as gauged by change in FS%. (**D**) WT% comparison between control and CDC groups. (**E**) Treatment effects as gauged by change in WT%. End‐point was consistent for all dogs and taken at the 3‐month time‐point.

**Figure 4:**
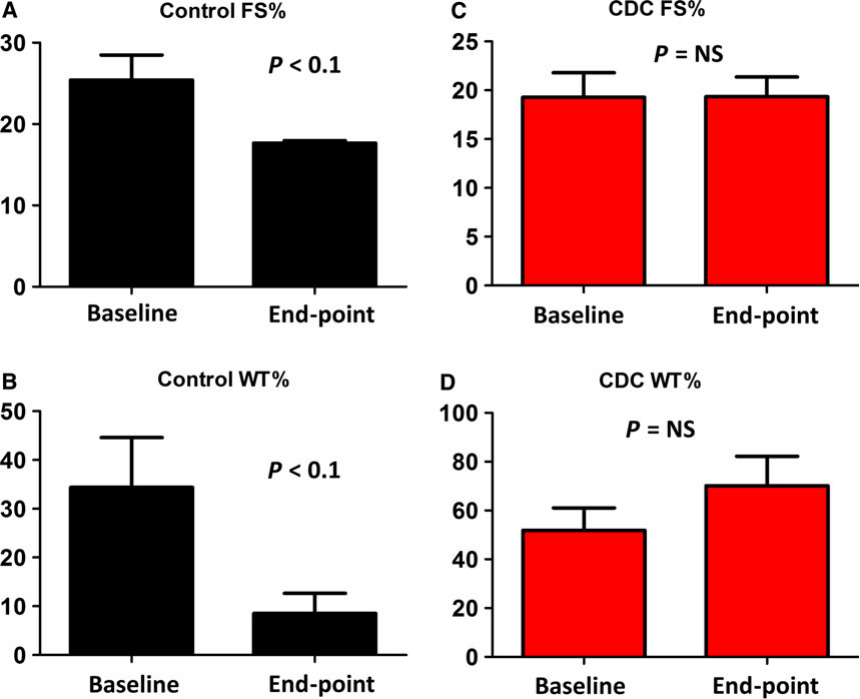
Intragroup analysis of FS% and WT% in control and CDC‐treated animals. (**A**) FS% at baseline and end‐point for the dogs in the control group. (**B**) WT% at baseline and end‐point for the dogs in the control group. (**C**) FS% at baseline and end‐point for the dogs in the CDC group. (**B**) WT% at baseline and end‐point for the dogs in the CDC group. End‐point was consistent for all dogs and taken at the 3‐month time‐point.

**Figure 5:**
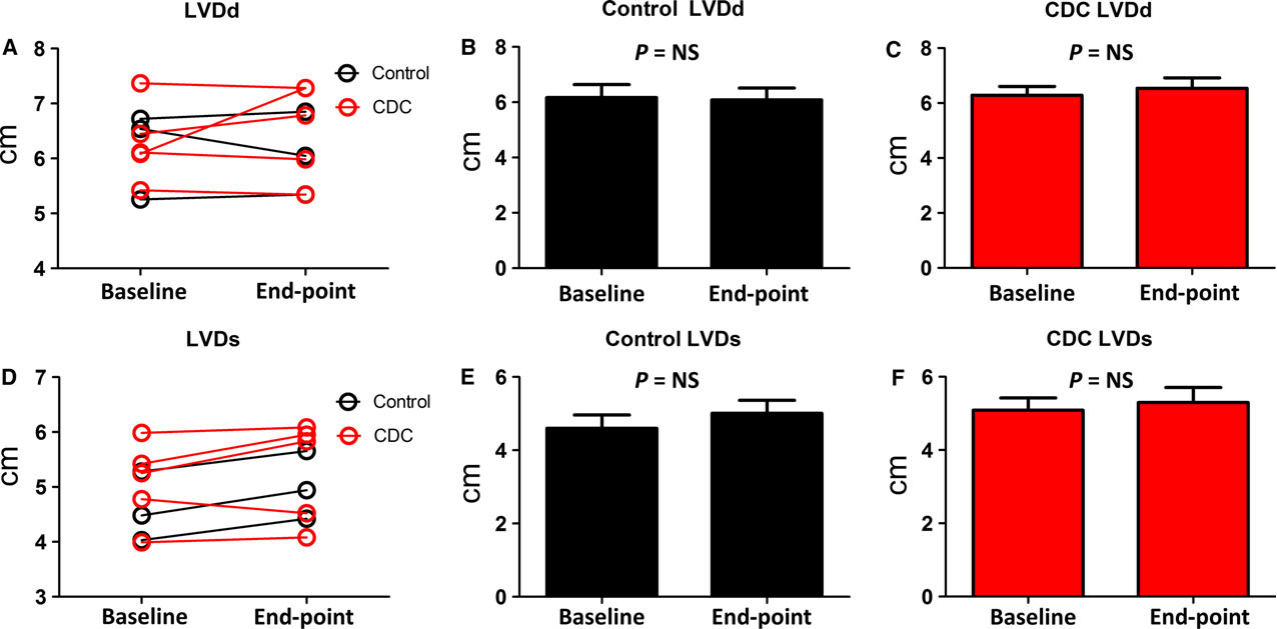
Left ventricle dimensions. (**A**) Comparison of LVDd change over the time between control and CDC groups. (**B**) LVDd at baseline and end‐point for the dogs in the control group (**C**) LVDd at baseline and end‐point for the dogs in the CDC group. (**D**) Comparison of LVDs change over the time between control and CDC groups. (**E**) LVDs at baseline and end‐point for the dogs in the control group. (**F**) LVDs at baseline and end‐point for the dogs in the control group. End‐point was consistent for all dogs and taken at the 3‐month time‐point.

**Figure 6:**
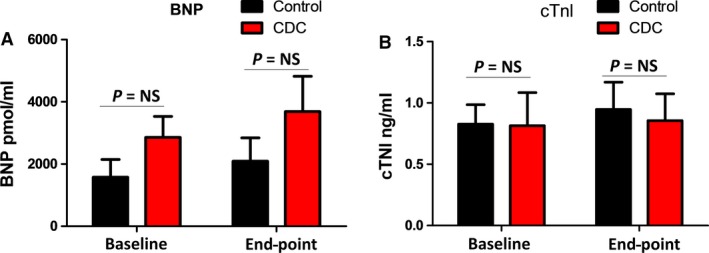
Cardiac failure and injury markers. (**A**) Comparison of serum BNP levels in control and CDC groups before and after infusion. (**B**) Comparison of serum cTNI levels in control and CDC groups before and after infusion. End‐point was consistent for all dogs and taken at the 3‐month time‐point.

Representative echocardiography image of a control and CDC‐treated animal at end‐point shows the wall motions of the heart (Fig. 3A). As an indicator of cardiac function, fractional shortening (FS%) continued to deteriorate in the control group (Fig. 3B, black lines). In contrast, CDC treatment robustly preserved FS% (Fig. 3B, red lines) with a combination of responders and non‐responders to the CDC treatment. Treatment effects were calculated as the change in FS% from the baseline to end‐point (Fig. 3C). A similar trend was found in per cent wall thickness, which was used as an another indicator of cardiac function (Fig. 3D and E). Intragroup analysis indicated decreases in FS% and WT% in the control group (Fig. 4A and B), while CDC treatment protected FS% and WT% (Fig. 4C and D). Left ventricular diameter diastolic (LVDd) and left ventricular diameter systolic (LVDs) were measured at baseline and end‐point (Fig. 5). In general, the changes in LV dimensions were indistinguishable between the two groups. The control and CDC treatment groups had similar brain natriuretic peptide (BNP) (Fig. 6A) and cardiac troponin I (cTnI) (Fig. 6B) levels at baseline and end‐point. This was measured to ensure the cell infusion did not exacerbate myocardial damage.

### Allogeneic CDC infusion does not engraft or cause immune rejection in the heart

**Figure 7:**
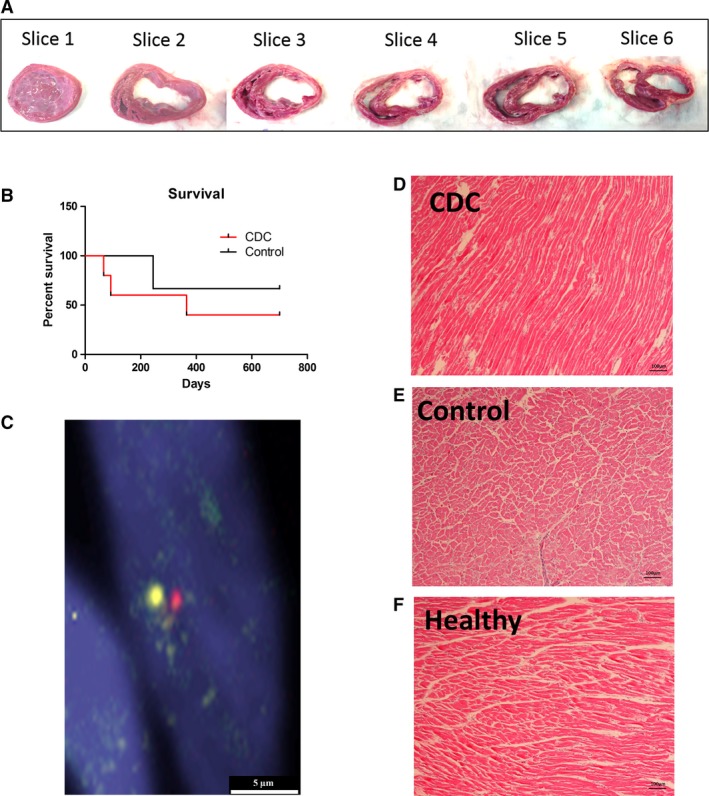
Heart histology. (**A**) Transversal section of CDC‐treated canine heart (**B**) Survival data comparing control (*n* = 3) and CDC‐treated (*n* = 5) groups. (**C**) Representative FISH stain showing lack of engraftment of infused male allogeneic cCDC in native female Doberman heart. (**D**) Haematoxylin and eosin staining showing no infiltration of cell nuclei from immune response, indicating allogeneic CDC infusion did not stimulate rejection. (**E**) A representative section from control animal heart. (**F**) A representative section from healthy Doberman heart. Scale bars = 5 and 100 μm

Autopsy on hearts from CDC‐treated, and control, dogs were sliced for gross inspection (Fig. 7A). Survival rates were similar for each group (Fig. 7B). No tumour formation was detected. Representative FISH staining (Fig. 7C) revealed no male donor cells in the female recipient heart. Haematoxylin and eosin staining shows the lack of concentration of cell nuclei (Fig. 7D), when compared to control heart (Fig. 7E), or a healthy (normal) heart (Fig. 7F), suggesting the allogeneic CDC infusion did not elicit an immune response from the heart. This was consistent from our previous findings in rat and human allogeneic CDC transplantations.

## Discussion

The last decade witnessed a burst of cell therapy trials for ischaemic cardiomyopathy. For the last 7 years, our laboratory has been studying CDCs and recently showed the regenerative potential of CDCs in a mouse model of induced DCM 20. Results from a recent clinical trial also indicated that infusion of autologous CDCs in patients with mild‐to‐moderate myocardial infarction reduced scar but increased viable tissue 8. A phase II clinical trial is ongoing to test the regenerative potential of allogeneic CDCs in patients with recent MI 21.

Domestic dogs suffer from a variety of spontaneously occurring heart diseases. In Doberman pinschers, the autosomal dominant inheritance pattern of DCM described in some families is similar to that of DCM in humans 7, 22. Expected survival time in Dobermans is strikingly short, and current treatment is symptomatic and palliative. This provides us with an excellent opportunity to use the Doberman pinscher as a naturally occurring model of DCM for CDC therapy.

In the present study, we demonstrate that cCDCs can be derived from adult dog hearts and that they are phenotypically similar to human and rodent CDCs (Fig. 1). Similar to human CDCs 20, cCDCs are positive for CD105 but have low expressions of CD90 and ckit. Recent studies have shown that ckit expression is irrelevant to the overall therapeutic benefit of CDCs, while CD90 expression undermines the regenerative potential of CDCs 23, 24. Allogeneic CDCs have proven to be safe in both rat models of myocardial infarction 25 and in an ongoing human trial 23, and the results of using allogeneic cCDCs derived from a Beagle dog donor for treating Doberman pinscher dogs with DCM appear to confirm this finding in dogs as well.

Before the cell infusion procedure, lot release criteria testing was performed to ensure the cell therapy product was free of bacterial agents, mycoplasma and endotoxin. In addition, we performed a storage stability assay to check for how long can the cells remained viable while being stored in the infusion solution at 4°C. Our data indicated that cell viability was >80% up to 5 hrs after collection (Fig. 2A), which provided abundant time for the myocardial distribution and infiltration of viable stem cells, as well as maintained secretion of growth factors for treatment. In addition, any normal delay in patient preparation for infusion would have little adverse effects on cell viability in clinical.

As DCM induces widespread myocardial dysfunction, it is essential to distribute large number of therapeutic stem cells globally. We designed a two‐vessel infusion protocol to cover the entire heart: 20 million cells into the left main artery and 10 million cells into the RCA (Fig. 2C and D). To ensure a fast and safe treatment, we devised a ‘wash–cell–wash’ infusion plan that allowed for an infusion to take less than 20 min. as well as ensuring no excessive cell accumulation in the vessel (Fig. 2B). The infusion scheme used here has also been applied to human patients. Previous studies have investigated the cardiac benefits of stem cells through intramyocardial injection in rodent models successfully 26, 27, but this method is invasive and limited to local areas of myocardium. Catheter‐based intra‐arterial infusion of stem cell therapies delivered at the time of myocardial reperfusion is emerging as a promising candidate to improve LV dysfunction in porcine model of MI 28, 29. In our present study, we applied intracoronary infusion by two vessels which involved left main artery and RCA, which we hope ensured the global distribution of therapeutic stem cells. In addition, delivering cells by ‘wash–cell–wash’ method may reduce the retention of stem cells in the coronary vessel, presumably promoting beneficial effects in remote areas of the heart.

DCM is characterized by progressive ventricular dilatation and contractile dysfunction. While there was no significant change in LVDd and LVDs from baseline to end‐point for both control and CDC groups (Fig. 5), our results revealed a consistent protection of FS% and WT% by the CDC therapy (Fig. 3). CDC therapy may also mitigate the progression of ventricular enlargement and deterioration of myocardial systolic function (Fig. 4). According to previous studies 10, 30, CDCs could promote cardiomyocyte proliferation and protect myocytes from antioxidant, antifibrotic and anti‐inflammatory injury. Cardiac stretch indicators (NT‐BNP) and cell injury markers (cTNI) showed no significant difference between the control and CDC groups (Fig. 6), suggesting cell infusion had no benefit, but also did not exacerbate myocardial stretch or damage as measured by these biomarkers.

Compared to autologous cell therapy, allogeneic cell products offer several benefits, namely the short time required for production and the low cost per dosage. In addition, patient‐specific tissue harvesting and cell processing may introduce possible variations in cell potency related to patient age and disease 31. One major concern of allogeneic therapy is the potential risk of host immune response. Previous studies have investigated the safety of allogeneic CDC transplantation in rats and pigs with myocardial infarction 25, 28, but the safety and efficacy of allogeneic CDCs in DCM models have not been explored. Here, we tested the specific hypothesis that allogenic CDCs could not engraft permanently in transplanted myocardium, but could still exert therapeutic effects. No immune cell infiltration was observed in the recipient heart (Fig. 7), which attested the safety of allogeneic cell therapy in the DCM model 25.It has been reported that there were no circulating antidonor antibodies after allogeneic CDC transplantation in rats with MI 25. In addition, the amount and volume of CDC therapy are relatively small compared to those in blood transfusion, and it has also been shown that injected CDCs do not persist beyond 3 weeks in the heart 25. Thus, the absence of such sensitization may explain why allogeneic cells could be survived in recipient. These results open the door to many different allogeneic treatments that can be tested in the future.

In summary, we derived cCDCs from adult dog hearts and showed their safety in an allogeneic canine DCM model.

## Limitations

There are some limitations to this study. The use of client‐owned animals that matched criteria for study limited the total number of animals that could be used. This limitation in sample size prevented our ability to find true significance in many factors that were trending towards significance. Another limitation is that only one cell donor was used for all cell recipients.

## Conflicts of interest

The authors confirm that there are no conflict of interests.
